# Successful living-donor liver transplantation for sustained liver failure even after resolution of infiltrative massive hepatic invasion of stage 4S neuroblastoma: a case report

**DOI:** 10.1186/s40792-023-01681-0

**Published:** 2023-06-08

**Authors:** Kanta Jobara, Ayako Yamamori, Masato Shizuku, Nobuhiko Kurata, Yasuhiro Fujimoto, Hideki Muramatsu, Yoshiyuki Takahashi, Yasuhiro Ogura

**Affiliations:** 1 Department of Transplantation Surgery, Nagoya University Hospital, 65 Tsurumai-Cho, Showa-Ku, Nagoya, 466-8560 Japan; 2 Department of Pediatrics, Nagoya University Graduate School of Medicine, Nagoya, Japan

**Keywords:** Stage 4S neuroblastoma, Liver failure, Living-donor liver transplantation

## Abstract

**Background:**

Neuroblastoma is the most common extracranial solid tumor in childhood. Stage 4S neuroblastoma is a unique subset of neuroblastoma characterized by a favorable course and potentially low malignancy with a high rate of spontaneous tumor regression. However, recent reports have shown that there is a subgroup of patients with stage 4S neuroblastoma characterized by *MYCN* amplification, chromosomal aberrations, age of < 2 months at diagnosis, and significantly poorer outcomes.

**Case presentation:**

A 1-month-old male infant with a huge abdominal tumor was transferred to our hospital and diagnosed with stage 4S neuroblastoma. The patient showed respiratory distress due to abdominal compartment syndrome secondary to massive hepatic invasion, and he required a silo operation and mechanical ventilation. After chemotherapy using carboplatin and etoposide, the infiltrative massive hepatic invasion was resolved and the abdominal compartment syndrome gradually improved; however, liver dysfunction as evidenced by hyperbilirubinemia, coagulopathy, and hyperammonemia continued. At the age of 3 months, living-donor liver transplantation was performed for treatment of sustained liver failure using a reduced lateral segment graft from the patient’s father. Post-transplant liver function recovered immediately. Examination of the explanted liver demonstrated that the majority of liver tissue had been replaced by fibroblastic cells after massive hepatocyte dropout. There were only small areas of residual neuroblastoma cells in the liver specimen. The patient was discharged from the hospital 5 months after transplantation with home intermittent respiratory support. At the time of this writing (23 months after liver transplantation), he was in good condition with no signs of recurrence of neuroblastoma.

**Conclusions:**

We have herein presented a case of successful pediatric living-donor liver transplantation for sustained liver failure even after resolution of infiltrative massive hepatic invasion of stage 4S neuroblastoma. Our case clearly shows that liver transplantation can be added as an appropriate extended treatment option for liver failure after resolution of stage 4S neuroblastoma.

## Background

Neuroblastoma is the most common extracranial solid tumor in childhood [1]. It originates from primordial neural crest cells and sometimes invades adjacent organs or exhibits distant metastasis. Most cases of neuroblastoma are treated by surgical resection and/or chemotherapy depending on the disease progression. However, there is a unique subset of neuroblastoma called stage 4S neuroblastoma, in which “S” stands for “special” [2]. The original definition of stage 4S neuroblastoma was a stage 1 or 2 primary tumor in a patient aged < 12 months with dissemination limited to specific sites, such as the liver, bone marrow (< 10% invasion), or skin. In 2009, the International Neuroblastoma Risk Group Staging System (INRGSS) extended the age limit of S4 neuroblastoma to 18 months, and the primary tumor stage was no longer taken into account (thus, stage 4S is similar to stage MS in the INRGSS) [3].

In general, stage 4S neuroblastoma is believed to be characterized by a favorable disease course and potentially low malignancy with a high rate of spontaneous tumor regression. Despite these optimistic characteristics of stage 4S neuroblastoma, its long-term survival rates are estimated to range from 65% to 92% [4, 5]. Notably, previous reviews revealed a poor prognosis in a minority of patients with stage 4S neuroblastoma aged < 2 months or with symptoms regardless of aggressive treatment, such as chemotherapy or tumor resection [6, 7]. Moreover, studies have revealed significantly poorer outcomes in a subgroup of patients with stage 4S neuroblastoma characterized by *MYCN* amplification, chromosomal aberrations (1p loss of heterozygosity, 11q aberration, 17q gain), diploidy, and age of < 2 months at diagnosis [1, 8–12].

We herein report a serious case involving a 3-month-old infant with stage 4S neuroblastoma who developed sustained liver failure and was treated by living-donor liver transplantation (LDLT) even after disease resolution of infiltrative massive hepatic invasion was achieved by chemotherapy.

## Case presentation

**Fig. 1:**
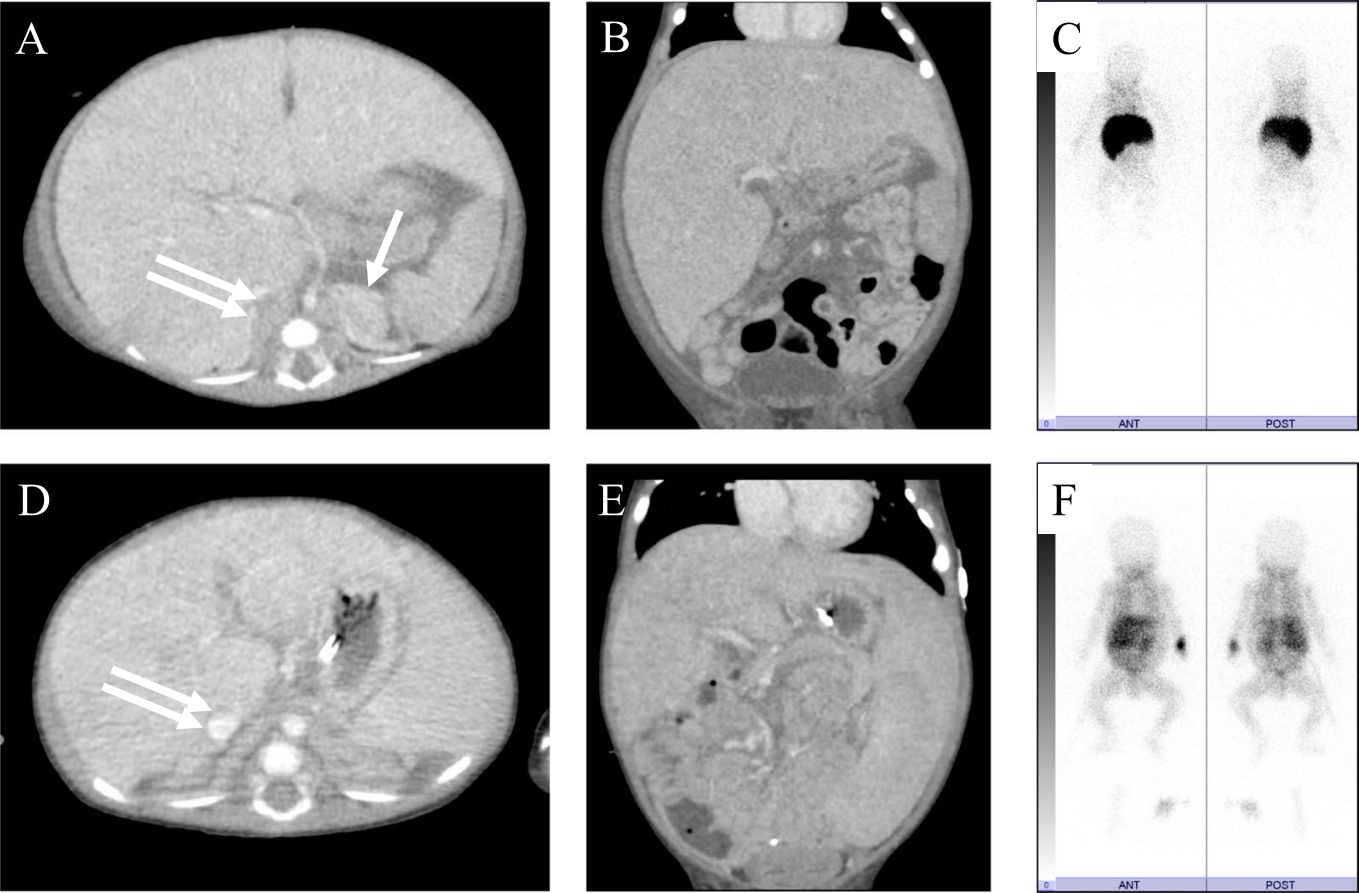
Enhanced computed tomography scan and iodine-123 metaiodobenzylguanidine scintigraphy on admission (**A–C**) and after chemotherapy (**D–F**). The single arrow indicates left adrenal gland swelling, and the double arrows indicate the compressed inferior vena cava by the enlarged tumorous liver. Note that the enlarged liver (**B**) changed to a normal size (**E**) after chemotherapy. **D** Left adrenal gland swelling disappeared and the inferior vena cava was relieved by improvement of the hepatomegaly. Iodine-123 metaiodobenzylguanidine scintigraphy on admission (**C**), significant uptake was seen in the liver, but minimal uptake was seen in the left adrenal gland. **F** After chemotherapy, the significant uptake in the liver disappeared

A 1-month-old male infant with a huge abdominal tumor was transferred to our hospital. The patient exhibited severe abdominal distension, an umbilical hernia, and severe edema of the lower extremities and scrotum. He required an oxygen mask because of respiratory distress. Computed tomography showed massive hepatomegaly with left adrenal gland swelling (Fig. 1A, andB). Iodine-123 metaiodobenzylguanidine (MIBG) scintigraphy demonstrated significant uptake in the liver but minimal uptake in the left adrenal gland (Fig. 1C). Laboratory data on admission showed severe liver dysfunction, as evidenced by hyperbilirubinemia (total bilirubin 10.1 mg/dL, direct bilirubin 2.5 mg/dL) and coagulopathy (PT-INR 1.67, APTT 46.70%, fibrinogen 99 mg/dL, AT-III 23.4%). Significant elevation of neuron-specific enolase (93.7 ng/mL), urine vanillylmandelic acid (499 μg/mg Cr), and urine homovanillic acid (378 μg/mg Cr) was also confirmed. Although bone marrow examination revealed negative cytology, further tests confirmed minimal residual disease, suggesting the existence of neuroblastoma cells in the bone marrow. An *MYCN* amplification test was negative, and chromosomal abnormalities including 11q deletion were not observed. We finally diagnosed the patient with stage 4S neuroblastoma.

**Fig. 2:**
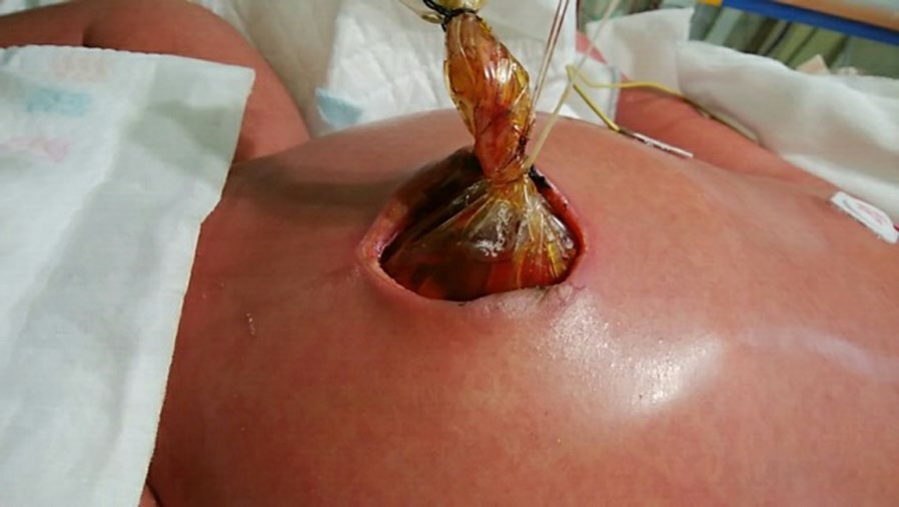
Postoperative photograph of silo operation (day #12). Because of gradual relief of abdominal compartment syndrome along with liver tumor resolution, the abdominal contents in the wound retractor were successfully returned into the abdominal cavity 12 days later

Although the stage 4S neuroblastoma was expected to have a high likelihood of spontaneous tumor regression, the patient’s abdominal compartment syndrome, hyperammonemia, and coagulopathy deteriorated. Seven days after the patient was transferred to our hospital, chemotherapy using carboplatin and etoposide was administered with expected acceleration of tumor regression compared with its natural course without chemotherapy, based on the Children’s Oncology Group A3961 low-/intermediate-risk course I regimen. However, because the patient developed respiratory failure secondary to abdominal compartment syndrome, which required intensive care unit admission with respiratory ventilation, a silo operation using an Alex wound retractor for the eviscerated intestine was performed to manage the severe abdominal compartment syndrome. The hepatomegaly was gradually relieved, and the silo was successfully closed 12 days postoperatively (Fig. 2). Partial disease resolution was also confirmed by computed tomography (Fig. 1D, E) and MIBG scintigraphy (Fig. 1F).

**Fig. 3:**
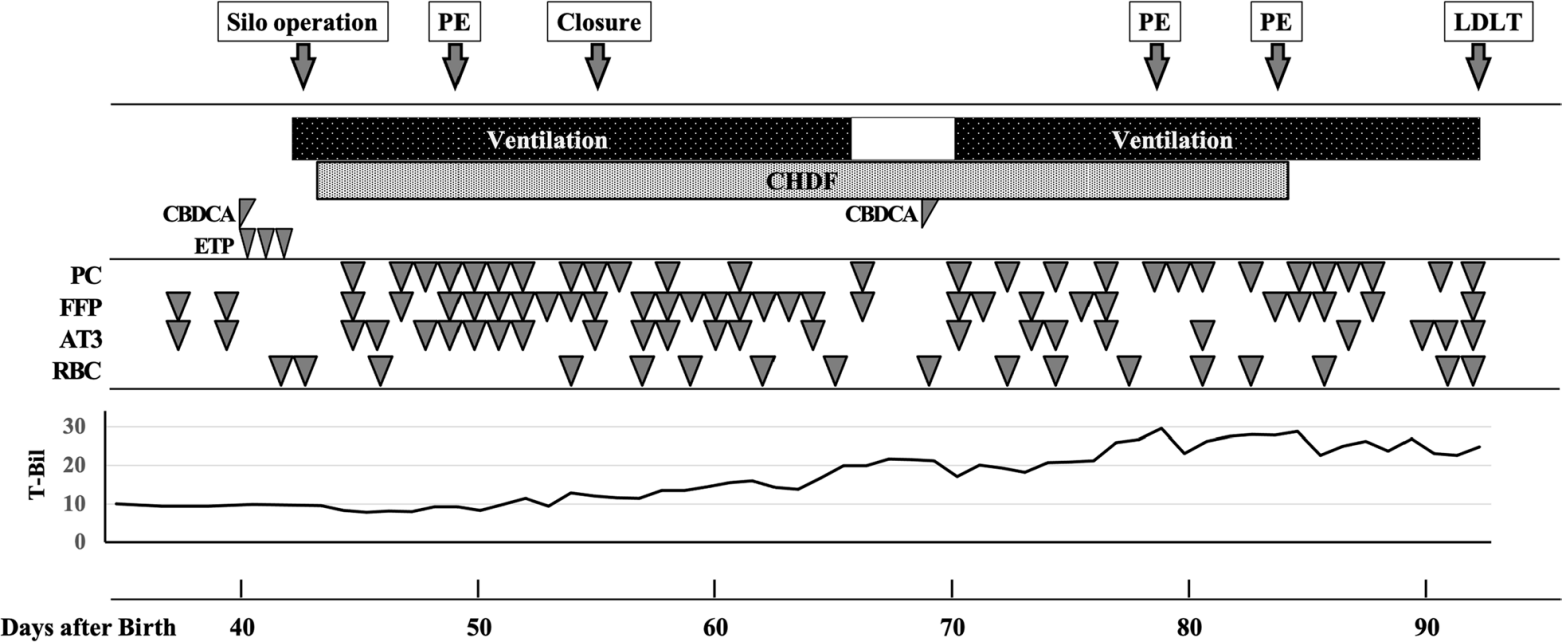
Clinical course from patient transfer to liver transplantation. Mechanical ventilation and liver support were required during this period. Blood transfusion and fractionated plasma products were administered repeatedly. Hyperbilirubinemia persisted until liver transplantation. *PE* plasma exchange, *LDLT* living-donor liver transplantation, *CHDF* continuous hemodiafiltration, *CBDCA* carboplatin, *ETP* etoposide, *PC* platelet concentrate, *FFP* fresh-frozen plasma, *AT3* anti-thrombin III, *RBC* red blood cells, *T-Bil* total bilirubin

Despite the decrease in tumor size, liver dysfunction continued. Fresh-frozen plasma, thrombocyte concentrates, and antithrombin III were administrated repeatedly, and continuous hemodiafiltration and plasma exchange were continued for liver support (Fig. 3).

**Fig. 4:**
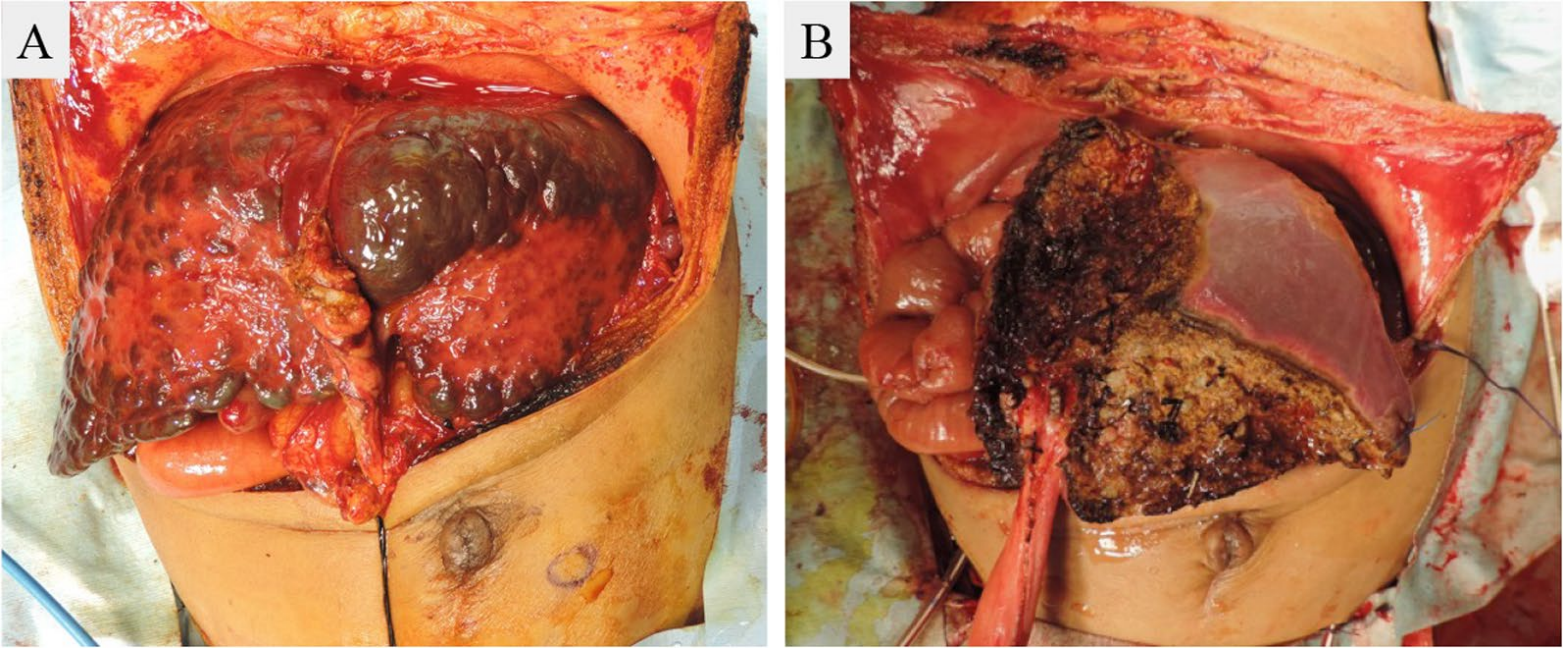
Intraoperative photograph during living-donor liver transplantation. **A** Native liver and **B** reduced lateral segment liver graft

**Fig. 5:**
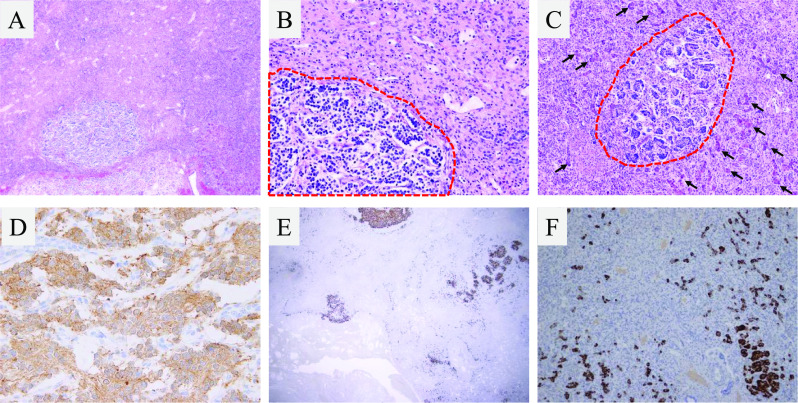
Histological findings of explanted liver. **A**–**C** Hematoxylin–eosin staining. Note the areas of small residual neuroblastoma cells (inside red line). After tumor regression, few scattered hepatocytes were present (black arrows), and the remaining areas were replaced by fibroblasts after hepatocyte dropout. Immunohistochemical staining with **D** synaptophysin and **E** neuron-specific enolase showed small residual neuroblastoma cells in vascular structures, and immunohistochemical staining with **F** hepatocyto paraffin 1 (HepPar-1) showed scattered hepatocytes (significant dropout of hepatocytes)

One month after patient transfer, the pediatric team referred the patient to the transplant team for consideration of liver transplantation. Although the levels of neuron-specific enolase, urine vanillylmandelic acid, and urine homovanillic acid as well as the imaging findings were improving, the liver failure persisted. While the patient’s father was evaluated to determine whether he could serve as a living donor, a wait-and-see approach was undertaken for the next 2 weeks to monitor for possible recovery of the patient’s liver function. Because of no sign of recovery of the patient’s liver function was observed, LDLT was performed at 93 days after birth. Because the recipient’s body weight at the time of transplantation was 4.7 kg, the lateral segment graft was reduced at the back table as well as in situ for graft accommodation (Fig. 4). Because left adrenal gland swelling as a primary lesion was already disappeared on the preoperative image study (Fig. 1D), left adrenal gland was not resected during transplant procedure. Macroscopically, the explanted liver was cholestatic and fibrotic. Hematoxylin–eosin staining and immunohistochemical microscopy showed massive dropout of hepatocytes, fibroblast replacement, and slight existence of residual neuroblastoma cells (Fig. 5A–F). The massive loss of hepatocytes was similar to that seen in fulminant hepatic failure, which explains this patient’s preoperative sustained liver failure status. Any sign of vascular complications including veno-occlusive disease were not identified clinically and microscopically.

After transplantation, the patient’s liver function and coagulation status recovered quickly. Due to the unique character of spontaneous regression ability of stage 4S neuroblastoma, no chemotherapy was considered postoperatively. Because of his long pre-transplant respiratory ventilation, he required a tracheostomy for long-term respiratory rehabilitation. He was discharged from the hospital 5 months after transplantation with home intermittent respiratory support. At the time of this writing (23 months after LDLT), he was in good condition with no signs of recurrence of neuroblastoma.

## Discussion

Stage 4S neuroblastoma is believed to be characterized by a favorable disease course, potentially low malignancy, and a high rate of spontaneous tumor regression [2]. However, recent studies have demonstrated unfavorable outcomes in patients with stage 4S neuroblastoma. In a study of cases from the Italian Neuroblastoma Registry, De Bernardi et al. [6] reported that symptomatic patients at the time of diagnosis had poorer outcomes than asymptomatic patients. The presence of any major symptom was associated with lower overall survival rates (77.4% for hepatomegaly, 59.5% for dyspnea, and 73.5% for organ dysfunction). Moreover, their multivariate analysis demonstrated that the hazard ratio of patients with hepatomegaly and dyspnea with/without organ dysfunction was as high as 24.1 compared with patients without major symptoms. Because our patient had hepatomegaly, dyspnea, and hepatic dysfunction, he was expected to have a very low chance of survival by normal treatment.

To date, only three case reports of liver transplantation for stage 4S neuroblastoma have been published worldwide. The first report involved emergent liver transplantation for treatment of severe coagulopathy in a patient with a pretransplant diagnosis of disseminated liver metastasis of infantile hepatic hemangioendothelioma [13]. However, the case was misdiagnosed preoperatively, and the explanted liver pathology revealed neuroblastoma. The second report involved a 2.5-month-old infant who had stage 4S neuroblastoma with liver metastases [14]. The indication for liver transplantation was chemotherapy-induced veno-occlusive disease leading to liver cirrhosis. The third case report involved a 30-day-old male infant who had stage 4S neuroblastoma with abdominal compartment syndrome and respiratory distress [15]. After confirmation of the diagnosis, the patient received chemotherapy and underwent multiple abdominal wall operations using a GORE–TEX patch to relieve pressure on the compromised abdomen before life-saving split liver transplantation at the age of 3 months. He required a second liver transplant 2 weeks after the first transplantation because of post-transplant hepatic failure, and it took 4 months to completely close the abdominal wall. Only in the third case report, liver transplantation was truly indicated for stage 4S neuroblastoma; i.e., the indications for liver transplantation in the third case were liver failure and abdominal compartment syndrome.

To our knowledge, the present report is the fourth published case report of a patient whose life was saved by liver transplantation and the first case by LDLT. However, the indication for transplantation in our case was purely liver failure, because the stage 4S neuroblastoma status was under resolution as confirmed by laboratory tests, MIBG scintigraphy, and examination of the explanted liver. Therefore, this is the first case report that the liver failure related to stage 4S neuroblastoma could be saved by liver transplantation. The mechanism of liver failure in this case was speculated that rapid tumor invasion to entire liver eliminated hepatocyte initially, and then massive hepatocyte dropout continued even after resolution of stage 4S neuroblastoma from the liver. Because the general nature of stage 4S neuroblastoma is optimistic, liver failure caused by stage 4S neuroblastoma after massive hepatic tumor resolution could be a good indication for liver transplantation, similar to acute liver failure. As we do not know the risk of recurrence after liver transplantation in this very special condition of post-resolution of 4S neuroblastoma, close follow-up is required.

## Conclusions

We have herein presented a case of successful pediatric LDLT for sustained liver failure even after resolution of infiltrative massive hepatic invasion of stage 4S neuroblastoma. Because stage 4S neuroblastoma is characterized by a high rate of spontaneous tumor regression, the treatment algorithm varies from a wait-and-see approach to chemotherapy and/or abdominal decompressive surgery. However, our case clearly shows that liver transplantation can be added to conventional multimodal treatment strategies as an appropriate extended treatment option for liver failure after resolution of stage 4S neuroblastoma.

## Data Availability

The data that support the findings of this study are available from the corresponding author upon reasonable request.

## Abbreviations

INRGSS: International Neuroblastoma Risk Group Staging System
LDLT: Living-donor liver transplantation
MIBG: Metaiodobenzylguanidine
